# Variant -and individual dependent nature of persistent *Anaplasma phagocytophilum *infection

**DOI:** 10.1186/1751-0147-52-25

**Published:** 2010-04-15

**Authors:** Erik G Granquist, Kjetil Bårdsen, Karin Bergström, Snorre Stuen

**Affiliations:** 1 Department of Production Animal Clinical Sciences, Section of Small Ruminant Research, Norwegian School of Veterinary Science, Sandnes, N-4325, Norway; 2 National Veterinary Institute, Uppsala 75189, Sweden

## Abstract

**Background:**

*Anaplasma phagocytophilum *is the causative agent of tick-borne fever in ruminants and human granulocytotropic anaplasmosis (HGA). The bacterium is able to survive for several months in immune-competent sheep by modifying important cellular and humoral defence mechanisms. Little is known about how different strains of *A. phagocytophilum *propagate in their natural hosts during persistent infection.

**Methods:**

Two groups of five lambs were infected with each of two *16S *rRNA gene variants of *A. phagocytophilum*, i.e. *16S *variant 1 which is identical to GenBank no M73220 and *16S *variant 2 which is identical to GenBank no AF336220, respectively. The lambs were infected intravenously and followed by blood sampling for six months. *A. phagocytophilum *infection in the peripheral blood was detected by absolute quantitative real-time PCR.

**Results:**

Both *16S *rRNA gene variants of *A. phagocytophilum *established persistent infection for at least six months and showed cyclic bacteraemias, but variant 1 introduced more frequent periods of bacteraemia and higher number of organisms than *16S *rRNA gene variant 2 in the peripheral blood.

**Conclusion:**

Organisms were available from blood more or less constantly during the persistent infection and there were individual differences in cyclic activity of *A. phagocytophilum *in the infected animals. Two *16S *rRNA gene variants of *A. phagocytophilum *show differences in cyclic activity during persistent infection in lambs.

## Background

*Anaplasma phagocytophilum *is an obligate intracellular bacterium, transmitted by *Ixodes *ticks, and is recognized as the causative agent of TBF (tick-borne fever) in sheep and HGA (human granulocytotropic anaplasmosis) in humans [1-3]. Estimates suggest that approximately 300.000 lambs are infected by *A. phagocytophilum *on pastures in Norway each year, inflicting considerable economic and animal welfare consequences [4,5]. It is suggested that tick-borne diseases and particularly anaplasmosis are underreported in veterinary and human medicine in Norway [6]. One reason for this underestimation might be attributed to the diversity in virulence and thus clinical manifestation among genetic variants of *A. phagocytophilum *[7,8]. Five *16S *rRNA gene variants of *A. phagocytophilum *have previously been identified to infect sheep in Norway, and one of these variants is known to cause severe clinical disease in domestic sheep (Variant 1) [5,9]. Different genetic variants have been found within the same flock of sheep and even within single animals [5].

By modifying important cellular and humoral defence mechanisms, *A. phagocytophilum *is able to survive and propagate for several months in immune-competent sheep [2,10,11], which may be crucial for the survival of the organisms due to the lack of transovarial transmission between generations of ticks [12,13]. Because of the brevity of acute *A. phagocytophilum *infection, transmission may rely on the tick's ability to acquire the organism from persistently infected sheep [14]. The ability for some *16S *rRNA gene variants to establish sustained and persistent bacteraemias may contribute to enhanced transmission from host and tick and favour the spread and propagation of certain genetic strains in nature [14,15].

The present study aims to investigate *in vivo *propagation of two naturally occurring sheep variants of *A. phagocytophilum *during persistent infection in lambs by qPCR (absolute quantitative real-time PCR).

## Materials and methods

### Experimental infection of lambs and blood sampling

Eleven 5-months-old lambs of the Norwegian Dala breed, were housed indoors before and during the experimental period lasting for 184 days. The experiment was approved by the National Animal Research Authority in Norway. Five lambs (L1-L5) were intravenously inoculated with 1 ml of a whole blood dimethyl sulphoxide (DMSO) stabilate of the *16S *rRNA gene variant 1 (Identical to GenBank no M73220), containing 9.03 × 10^7 ^*A. phagocytophilum *organisms ml^-1^. Another five lambs (L6-L10) were inoculated with 1 ml whole blood DMSO stabilate of the *16S *rRNA gene variant 2 (Identical to GenBank no AF336220), containing 1.6 × 10^8 ^*A. phagocytophilum *organisms ml^-1^. The inocula had been stored as heparinised blood with 10% DMSO at -75°C, and had earlier been used in several inoculation trials [15]. The infection doses were quantified according to the protocol described under materials and methods section "DNA isolation and qPCR analysis of *A. phagocytophilum *infection". One lamb (L11) was kept as an uninfected control. All lambs were examined and found negative for *Mycoplasma ovis *(formerly *Eperythrozoon ovis*) infection by blood smear analysis prior to inoculation. EDTA blood samples were collected from the jugular vein on day 0 (before infection), day 3 (post infection) and then every second or third day for a six month period. Blood samples were stored at -75°C for later PCR analysis. In addition, total and differential neutrophil counts were determined electronically (ADVIA, Bayer) for the first 24 days of the infection. Serum samples were collected in week 0, 2, 4, 8, 12, 16, 20 and 26 of the experimental infection. The rectal temperature was recorded before each blood sampling and the incubation period was defined as the number of days between inoculation and the first day of fever (> 40°C). The duration of fever was recorded as the number of days with fever [16].

### DNA isolation and qPCR analysis of *Anaplasma phagocytophilum *infection

An automated isolation procedure based on magnetic bead technology was performed by the application of the MagNA Pure LC instrument (Roche) and the MagNA Pure LC DNA Isolation Kit I Blood Cells High Performance (Roche). Briefly, a total number of 81 EDTA blood samples from each of the infected animals were thawed at room temperature and 200 μl blood was transferred to the DNA isolation procedure according to the instruction manual (Roche). The isolated DNA was eluted with 100 μl low salt buffer and stored at -20°C awaiting PCR analysis. The concentration of DNA in each sample was determined by OD_260 _spectrophotometry (GeneQuant II, Pharmacia Biotech, Cambridge, UK). The samples were diluted 1:100 before PCR analysis.

A 275 bp plasmid was designed from the N-terminal conserved part of the *msp2(p44) *expression site for absolute quantification against a standard curve. The plasmid represents a region between 364 and 112 bps upstream of the highly conserved 5' sequence that encodes the LAKT amino acid residue which flanks the N-terminal end of the hyper variable region in the *msp2(p44) *expression site (Table 1) [17]. The primers were ApMSP2252 5' ACAGTCCAGCGTTTAGCAAGA and ApMSP2 459 5' CACCACCAATACCATAACCA amplifying a product of 208 bp covering both the plasmid and the conserved N-terminal region of the expression site for *msp2(p44) *(Table 1). Plasmid and primers were manufactured by TIB Molbiol (Germany). The plasmid was included as duplicates in every PCR run at 10 fold dilution series ranging from 10^1 ^to 10^6 ^copies. A Light Cycler 480 instrument (Roche) was used for the real-time absolute quantification PCR analysis. 96 well white plates were loaded with a reaction mix consisting of 1 μl (10 μM) ApMSP2252 primer, 1 μl (10 μM) ApMSP2459 primer, 3 μl RNAse free H_2_O, 10 μl LightCycler 480 DNA SYBR Green I Master and 5 μl sample. Plates were sealed by sealing foil and centrifuged at 1200 rpm for two minutes. Samples and non-template controls were run in triplicates on each plate.

**Table 1:** Primer -and plasmid sequences for qPCR of the *A. phagocytophilum msp2 (p44) *expression site (partial sequence).

Primer	Sequence	Conc.	Tm °C
ApMSP2252	5'ACAGTCCAGCGTTTAGCAAGA-3'	0.5 *μ*M	57.0

ApMSP2459	5'GCACCACCAATACCATAACCA-3'	0.5 *μ*M	56.5

**Plasmid construct**			

5'GAATTCGCCCTT**ACAGTCCAGCGTTTAGCAAGA**TAAGAGATTTTAGTA TAAGGGAGAGTAACGGAGAGACTAAGGCAGTATATCCATACTTAAAGGA TGGAAAGAGTGTAAAGCTTGAGTCTAACAAGTTTGACTGGAACACTCCTG ATCCTCGGATTGGGTTTAAGGACAACATGCTTGTAGCTATGGAAGGCAGT GT**TGGTTATGGTATTGGTGGTGC**CAGGGTTGAGCTTGAGATTGGTTACG AGCGCTTCAAGACCAAAAGGGCGAATTCT-3'			

Primer binding sites are indicated (underscored) in the sequence of the plasmid construct. The concentrations of primers are calculated for the final reaction mix.

**Figure 1:**
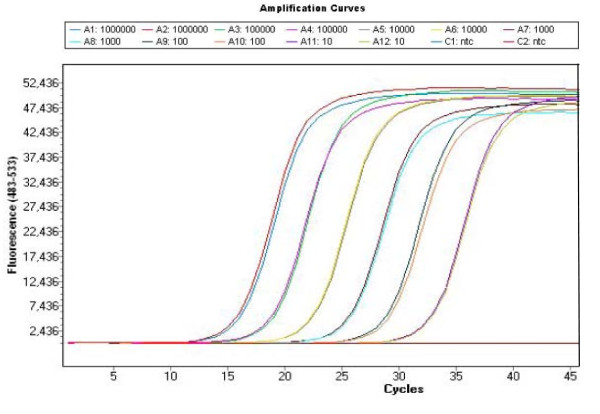
**qPCR amplification of the plasmid dilution series**. A plasmid dilution series was produced containing 10^1 ^to 10^6 ^copies of the *msp2 (p44) *gene of *A. phagocytophilum*. The figure shows the amplification cycles of the dilution series used as standard curve for quantification of the infection. In addition the figure shows the lack of amplification of the non template controls.

**Figure 2:**
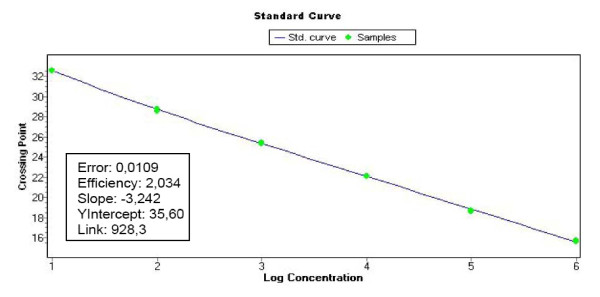
**Standard curve of the plasmid dilution series**. The standard curve was used for the quantification of *A. phagocytophilum *organisms in the blood from infected lambs.

**Figure 3:**
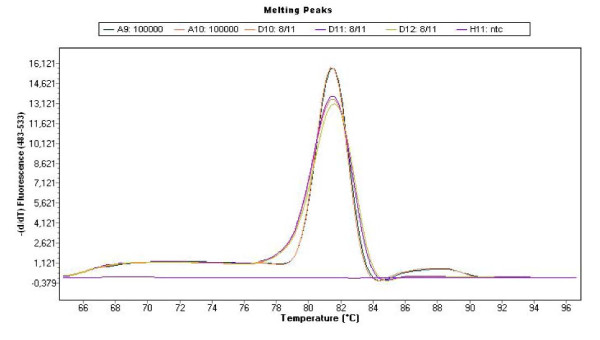
**Melting point analysis of the amplicons**. The figure shows the melting point analyses (Tm) of the plasmid, *A. phagocytophilum *organisms isolated from blood and non template controls. No primer dimers were formed.

**Figure 4:**
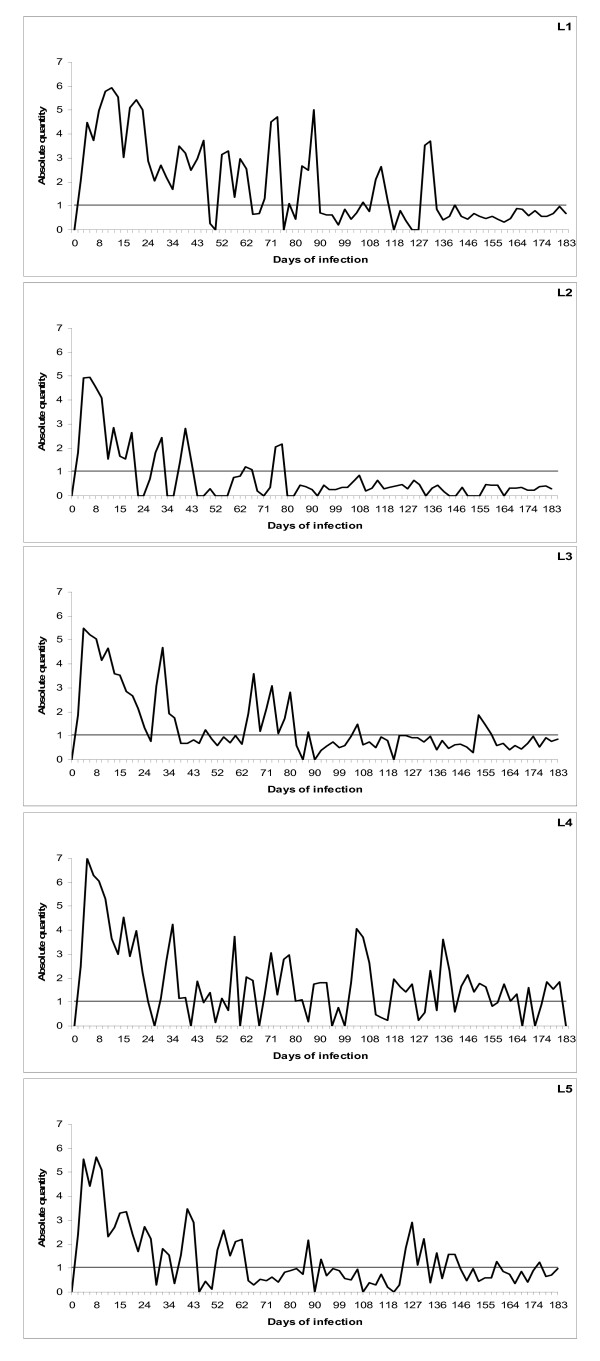
**qPCR of infection with *16S *rRNA gene variant 1 of *A. phagocytophilum***. Cyclic bacteraemia of persistent *A. phagocytophilum *infection in five lambs (L1-L5) inoculated with the Norwegian *16S *rRNA gene variant 1, monitored by qPCR for six months. The horizontal line shows the threshold for bacteraemia and represents the lowest (10 copies) plasmid dilution for the standard curve calibration. The results are presented as logarithm transformed means of triplicate C_q _readings (X) for each sample, calculated as Log_10 _(1+X).

**Figure 5:**
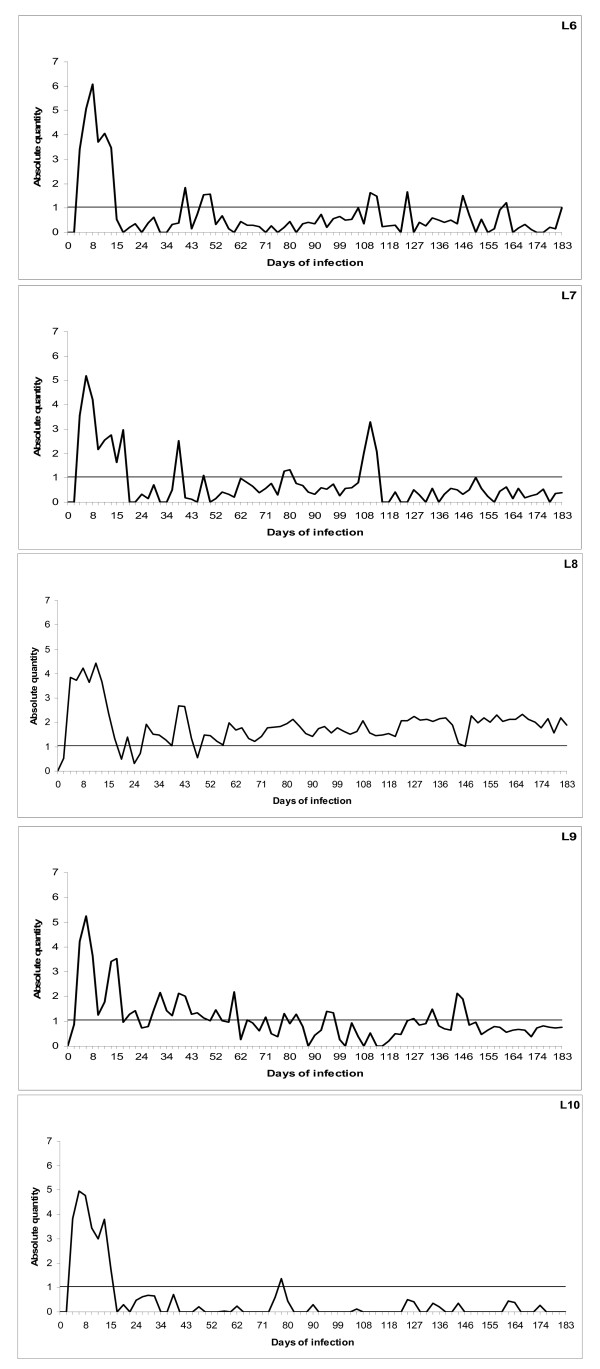
**qPCR of infection with *16S *rRNA gene variant 2 of *A. phagocytophilum***. Cyclic bacteraemia of persistent *A. phagocytophilum *infection in five lambs (L6-L10) inoculated with the Norwegian *16S *rRNA gene variant (GenBank no) AF336220 monitored by qPCR for six months. The horizontal line shows the threshold for bacteraemia and represents the lowest (10 copies) plasmid dilution for the standard curve calibration. The results are presented as logarithm transformed means of triplicate C_q _readings (X) for each sample, calculated as Log_10 _(1+X).

**Figure 6:**
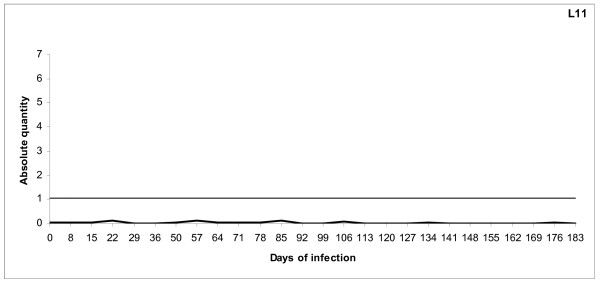
**qPCR of uninfected control animal (L11)**. The uninfected control animal was monitored by qPCR as described for the infected lambs throughout the experimental period. The horizontal line shows the threshold for bacteraemia and represents the lowest (10 copies) plasmid dilution for the standard curve calibration. The results are presented as logarithm transformed means of triplicate C_q _readings (X) for each sample, calculated as Log_10 _(1+X).

The standard curve was made from the mean of duplicate readings of the plasmid amplification. The quantification against the standard curve was automatically done using the LightCycler 480 Software (release 1.5.0) (Roche) (Fig. 1 and 2). The C_q _values (quantification cycle) were determined by the 2^nd ^derivative maximum method, and quantitative data (X) were estimated as the mean of triplicate C_q _values divided by the concentration (μg/ml) of DNA in each sample to correct for the differences in the efficacy of DNA preparations. Triplicate readings with mean values below 1 were regarded as zero and the amplicons were verified by melting point analysis (Tm) (Fig. 3). The quantitative data are presented as Log_10 _(1+X) on a linear graph for the longitudinal development of bacteraemia over time. A threshold was set at the lowest plasmid dilution (10^1 ^copies) and calculated as follows (Log_10 _(1+10^1^) = 1.04) to better distinguish the periods of bacteraemias (Fig. 4, 5 and 6).

### Serology

**Figure 7:**
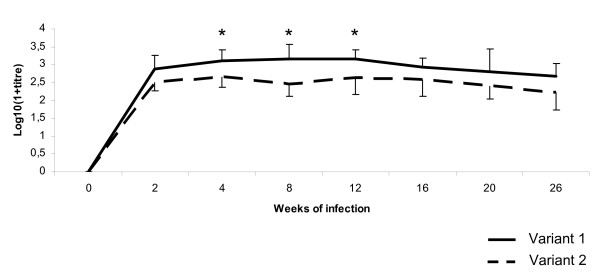
**Serology of *A. phagocytophilum *infection**. Mean antibody titre ± SD in lambs inoculated with *16S *rRNA gene variant 1 (continuous line) and *16S *rRNA gene variant 2 (dotted line) of *A. phagocytophilum *during a six months infection period. * P < 0.05.

Briefly, an indirect immunofluorescence antibody assay (IFA) was used to determine the developing polyvalent antibody titres to *A. phagocytophilum *in serum from the infected lamb. Two-fold dilutions of sera were added to slides pre-coated with antigen obtained from horses (formerly *Ehrlichia equi*) (Protatek, St. Paul. Minn.). Bound antibodies were visualized by fluorescein-isothiocyanate (FITC)-conjugated rabbit-anti-sheep immunoglobulin (Cappel, Organon Teknika, West Chester, PA). Sera were screened for antibodies at dilution 1:40. If positive, the serum was further diluted and retested. A titre of 40 or more was regarded as positive [18]. The titres were presented as Log_10_(1+titre) (Fig. 7).

### Statistics

For statistical analyses, two-sample t-tests were performed in the Statistix, version 4.0 (Analytical software). P values < 0.05 (two-tailed) were regarded as significant.

## Results

### Clinical manifestation and haematology

All lambs reacted with fever (> 40.0°C) within five to six days after inoculation. Table 2 summarizes the main clinical and haematological parameters during the acute phase of the infection.

**Table 2:** Mean ± SD values of clinicalvariables in the two groups of lambs inoculated with each of the two *16S *rRNA gene variants of *A. phagocytophilum*, variant 1 and 2 respectively.

*16S *gene variants of *A. phagocytophilum*	N	Incubation period (days)	Maximum fever (°C)	Duration of fever (days)	Nadir of neutropenia	Duration of neutropenia (days)
1	5	5.6 ± 0.89	41.8 ± 0.23**	9.4 ± 3.21**	0.29 ± 0.108*	6.3 ± 3.20*

*2*	5	6.6 ± 0.55	41.0 ± 0.34	2.2 ± 0.45	0.73 ± 0.236	0.3 ± 0.50

The incubation period is the number of days from inoculation until the first day of fever. Duration of fever is the number of days with fever during the infection. Nadir of neutropenia (< 0.7 × 10^9 ^Litre^-1^) is the lowest neutrophil count. * P < 0.05, ** P < 0.01.

### qPCR amplification of the *msp2/p44 *expression site

The lambs in both groups developed acute infections with detectable levels of bacteraemia by PCR within three days after inoculation with *16S *rRNA gene variant 1 and within three to five days with variant 2. The initial periods of bacteraemia had a mean duration of 25.6 (± 6.96) days with *16S *rRNA gene variant 1 and 11.4 (± 1.28) days with *16S *rRNA gene variant 2, respectively (P = 0.02) (Fig. 4 and 5). The initial bacteraemias were followed by lower cyclic levels of bacteraemia in all animals at individual, -and variant dependent frequencies and amplitudes. *16S *rRNA gene variant 1 cycled more frequently compared with *16S *rRNA gene variant 2 and had more periods of bacteraemia above threshold (mean of 10 periods compared to 6.2 periods respectively) (Fig. 4 and 5). The number of organisms detected in the blood was higher in periods of bacteraemia with variant 1 during the persistent phase of infection (Fig. 4). One lamb (L1) had exceptionally high numbers of organisms during the periods of bacteraemia (Fig. 4). In contrast, another lamb (L10) had low numbers of organisms and showed very little cyclic activity during the persistent phase with only one period of bacteraemia (Fig. 5). Lamb 8 (L8) had even levels of cyclic activity with very few days below threshold (10 organisms), during the persistent infection (Fig. 5). The uninfected control lamb (L11) was negative during the whole experimental period (Fig. 6).

### Serology

All lambs, except one (L7), seroconverted before day 17 after inoculation. This lamb did not have positive titre until day 59. Mean titres were higher for the lambs infected with *16S *rRNA gene variant 1 than lambs infected with variant 2 (Fig. 7).

## Discussion

In the present study, differences in clinical manifestation between two *16S *rRNA gene variants of *A. phagocytophilum *were observed during the acute phase of the infection. Lambs infected with gene variant 1 had a longer period of fever, significantly longer periods of initial bacteraemia, and a more severe neutropenia compared to lambs infected with gene variant 2. These results are in accordance with earlier observations [8]. Earlier studies have also reported that gene variant 1 of *A. phagocytophilum *is involved in the majority of fatal TBF cases in sheep [19].

It has previously been confirmed that *A. phagocytophilum *can establish long term infections in immune-competent sheep [2,20-22]. During this experiment, both *16S *rRNA gene variants persisted for at least six months, although the level of bacteraemia gradually diminished towards the end of the experiment (Fig. 4 and 5). Both variants showed a cyclic pattern with numbers of organisms waxing and waning at different frequencies, indicating that both individual and variant dependent differences exist. Periods of bacteraemia terminated by sharp reductions in number of organisms as previously described for the closely related organism, *Anaplasma marginale *[23,24]. This was hypothesized to reflect an antigen variant-specific immune response to *A. phagocytophilum *[25]. *A. marginale *seems to cycle regularly at approximate intervals of 5-8 weeks during persistent bacteraemia [14,23]. However, the present study indicates a much more frequent and inconsistent cyclic activity of *A. phagocytophilum*.

Transovarial transmission of *A. phagocytophilum *has been reported in certain tick species [26]. However, the maintenance of *A. phagocytophilum *within *I. ricinus *tick populations is unlikely, due to lack of evidence that transovarial transmission of *A. phagocytophilum *in female ixodid ticks occurs [13,27]. For the pathogens to survive in an *Ixodes*-host cycle, the duration of host infectivity must span the gap between seasons of tick activity [28].

Ixodid ticks normally remain attached for days to weeks depending on for instance tick species and their developmental stage [29]. The majority of the blood meal and thus host to tick transmission mainly occurs during the rapid feeding phase at the end of the attachment period [29], while tick to host transmission takes place during the first or second day after attachment in the preparatory feeding phase [30]. As lambs infected with variant 1 had more frequent cycles and higher number of circulating organisms than variant 2 for prolonged periods during the persistent infection, ticks are more likely to be exposed to this variant during feeding in the persistent period [14]. This may favour the spread of this variant in nature and explain why gene variant 1 is more frequently encountered than gene variant 2 by diagnostic sampling in randomly selected sheep flocks [5]. However, this statement has to be further elucidated in experimental transmission trials using ticks. For instance, the transmission efficacy of gene variant 1 and 2 in *Ixodes *ticks, respectively, are unknown and requires further investigation. Previous studies have shown that the efficacy of transmission is influenced by the number of circulating neutrophils and possibly the number of feeding ticks [31]. The ticks also seem to promote leukocyte trafficking to the tick bite site, thus directly influencing the efficacy of transmission [32].

The cyclic behaviour of *A. phagocytophilum *during persistent infection may be influenced by the immunological response towards changing surface proteins as previously suggested, although different intrinsic immunogenic properties may be carried by the various *16S *rRNA genotypes [10,25,33]. The stronger immune response with variant 1 than variant 2 in the present study may indicate differences in immune stimulatory potential between the variants [5], differences in individual humoral responses or higher antigenic relatedness of variant 1 with the used test antigen.

In the present study, there were marked differences between individual animals with respect to the amplitude of bacteraemia, number of bacteraemia periods, time of serological conversion -and response regardless of genetic variant. The reason for this strong individual variation is however unknown, and has to be further elucidated.

## Conclusion

Two naturally occurring sheep strains of *A. phagocytophilum *established persistent infections for at least six months. Both variants performed cyclic bacteraemia, but variant 1 showed more frequent bacteraemias and higher number of organisms during the infection. In the present study, organisms were available from blood more or less constantly during the entire study period. Future studies should use ticks to investigate whether the transmission efficacy is different among gene variants of *A. phagocytophilum *and determine if ticks are able to take up organisms continuously during the persistent phase. The ability of *A. phagocytophilum *to establish long term persistence combined with a large repertoire of genetic variants make the movement of sheep between flocks and geographical locations likely to introduce novel genetic variants to new areas.

## Competing interests

The authors declare that they have no competing interests.

## Authors' contributions

EGG performed the quantification PCR and drafted the manuscript. KBa assisted in interpretation of quantification data. KBe performed the serology. SS designed -and supervised the study and performed the statistical calculations. All authors have read and approved the final manuscript.
